# Social Avoidance and Social Adjustment: The Moderating Role of Emotion Regulation and Emotion Lability/Negativity Among Chinese Preschool Children

**DOI:** 10.3389/fpsyg.2021.618670

**Published:** 2021-03-15

**Authors:** Jingjing Zhu, Bowen Xiao, Will Hipson, Chenyu Yan, Robert J. Coplan, Yan Li

**Affiliations:** ^1^Department of Preschool Education, Shanghai Normal University, Shanghai, China; ^2^Department of Psychology, Carleton University, Ottawa, ON, Canada; ^3^Shanghai Normal University, Tianhua College, Shanghai, China

**Keywords:** emotion regulation, social avoidance, emotion lability, China, social adjustment

## Abstract

The present study explored the role of emotion regulation and emotion lability/negativity as a moderator in the relation between child social avoidance and social adjustment (i.e., interpersonal skills, asocial behavior, peer exclusion) in Chinese culture. Participants were *N* = 194 children (102 boys, 92 girls, *M*_*age*_ = 70.82 months, SD = 5.40) recruited from nine classrooms in two public kindergartens in Shanghai, People’s Republic of China. Multi-source assessments were employed with mothers rating children’s social avoidance and teachers rating children’s emotion regulation, emotion lability/negativity and social adjustment outcomes. The results indicated that the relations between social avoidance and social adjustment difficulties were more negative among children lower in emotion regulation, but not significant for children with higher emotion regulation. In contrast, the relations between social avoidance and social adjustment difficulties were more positive among children higher in emotion lability/negativity, but not significant for children with lower emotion lability/negativity. This study informs us about how emotion regulation and emotion lability/negativity are jointly associated with socially avoidant children’s development. As well, the findings highlight the importance of considering the meaning and implication of social avoidance in Chinese culture.

## Introduction

Whether in the classroom or on the playground, it is not uncommon to see some children displaying socially withdrawn behaviors (Rubin et al., 2003). For example, almost 90% individuals reported that they have experienced shyness and withdrawal behavior in their lives (Zimbardo, 1977). Social withdrawal is a multi-dimensional construct that includes several motivational substrates, such as shyness, unsociability, and social avoidance (Asendorpf, 1990; Rubin et al., 2009). For example, some children experience *shyness* in social situations and withdraw to reduce feelings of wariness and anxiety, despite wanting to interact with others. Yet other children would be considered *unsociable* as they withdraw, not out of social anxiety, but out of a desire to spend time alone. *Social avoidance*, which is the combination of a desire for spending time alone and actively seeking to avoid social interaction (Asendorpf, 1990). Accordingly, shyness, unsociability, and social avoidance are characterized as related but distinct constructs and, therefore, it necessary to control for any shared variance to explore their unique effects and implications (Coplan et al., 2018). Social avoidance is concurrently and predictively associated with a number of maladaptive adjustment outcomes, such peer problems, social anxiety, and depression (Bowker et al., 2012; Coplan et al., 2013, 2018; Nelson, 2013).

Notwithstanding, the links between social avoidance and adjustment difficulties among children in Western societies, there is also evidence that the meaning and implications of social withdrawal vary as a function of cultural values (Chen et al., 2006). For instance, in China, avoiding social interaction violates deeply embedded social norms regarding group harmony and cohesion (Chen et al., 2009). Specifically, children who display unsociable behavior (preference for solitude) and avoid social interaction on purpose may be perceived as selfish and deviant (Chen et al., 2010; Ding et al., 2015). Thus, Chinese children who are socially avoidant may therefore be at risk of being ostracized and disliked by peers. Although previous studies have demonstrated that social emotional factors could moderate the relation between shyness, unsociability and adjustment outcomes (e.g., Rubin et al., 1995; Coplan et al., 2001; Hipson et al., 2019), little is known about the moderated role of those factors in the relation between social avoidance and adjustment difficulties. Specifically, not all socially avoidant children experience adjustments difficulties later on (Penela et al., 2015). It is possible that certain socio-emotional facts may either mitigate or exacerbate these outcomes among socially avoidant Chinese children. Thus, the primary goal of the present study was to address this gap by examining potential moderating roles of emotion regulation and emotion lability/negativity in the associations between social avoidance and adjustment difficulties among Chinese children.

Social avoidance is a distinct subtype of social withdrawal that involves a desire to avoid social interaction due to anxiety and a preference to spend time alone (Asendorpf, 1990). These two components, social anxiety and preference for solitude, jointly increase the likelihood of social maladjustment among these children. For example, feelings of social anxiety inhibit positive social interactions and reduce social opportunities, further contributing to problems in interpersonal relations (La Greca, 2001; Russell et al., 2011). Moreover, because socially avoidant children seek out more solitude, they might miss out on important opportunities to practice and develop new cognitive and social skills (Coplan et al., 2009; Jones et al., 2015). Indeed, past research has shown that compared to their more sociable counterparts, socially avoidant children experience more peer difficulties and internalizing problems. For example, Coplan et al. (2006) found that compared with other children, avoidant children reported the highest levels of negative affect and depressive symptoms and the lowest levels of positive affect and overall well-being. Similarly, Nelson (2013) reported that social avoidance was related to peer difficulties among young adults. Bowker and Raja (2011) also found that social avoidance was associated with peer-exclusion and loneliness among adolescents. In the present study, we focused on three social adjustment behaviors (e.g., asocial behavior, peer exclusion and interpersonal skills) which reflect children’s performance in social interaction. Asocial behavior refers to a low social approach motion and leads to one becoming isolated (Ladd and Profilet, 1996). Peer exclusion involves rejection from individuals or peer group (Ladd and Profilet, 1996). Interpersonal skills defined as cooperation with others, help others and sharing (Gresham and Elliott, 1990). These three components are widely used as the indices of social adjustments (Sette et al., 2017; Ooi et al., 2018; Hipson et al., 2019).

As mentioned before, the meaning and implications of different types of social withdrawal vary by cultural context (Chen et al., 2006). In Chinese culture, maintaining group harmony is an essential concern for individuals (Chen et al., 2010). Thus, removing oneself from the peer group and avoiding social interaction is viewed as selfish (Chen et al., 2009). This serves to make social avoidance a salient predictor of peer problems throughout Chinese children’s development (Ding et al., 2015; Sang et al., 2018). For instance, Ding et al. (2015) reported that hypothetical avoidant peers were viewed most negatively by Chinese children compared with described shy, unsociable children. Similarly, Coplan et al. (2016) found that Chinese socially avoidant children reported higher internalizing problems (i.e., depression, social anxiety, and loneliness) as compared to other children. More recently, Sang et al. (2018) reported that Chinese early adolescents’ social avoidance was associated with teacher-rated negative emotion and peer problems.

As discussed above, certain socio-emotional facts may protect socially avoidant children from experiencing adjustments difficulties. In the present study, we focused on emotion lability/negativity and emotion regulation. Given that those two factors are critical in promoting positive social interaction and coping with challenging and stressful situations, which has an impact on children’s personality development and their ways of interacting during social and close relationships (e.g., Kim-Spoon et al., 2013). Researchers tend to view emotion regulation as both a process that evolves over time, but also as a relatively stable temperamental trait (Diaz and Eisenberg, 2015). Two interrelated dimensions of temperament relevant to the process of emotion expression are lability/negativity and regulation. Lability/negativity corresponds to characteristic differences in how strongly and negatively children tend to react to emotion-eliciting events (Rothbart et al., 2004; Rothbart and Bates, 2006; Kim-Spoon et al., 2013). Children who are more emotionally labile tend to be less socially competent and exhibit more peer problems (Eisenberg et al., 1993; Stringaris and Goodman, 2009). In contrast, regulation corresponds to how quickly children can decrease their emotional arousal (e.g., downregulate negative emotions). Children higher in regulation have better interpersonal skills and tend to be higher in prosocial behavior and, interpersonal sensitivity, social competence and peer preference (Calkins et al., 1999; Halberstadt et al., 2001; Eisenberg et al., 2006; Blair and Raver, 2015; Song et al., 2018). For example, Hipson et al. (2019) found that preschool children’s active regulation was negatively associated with asocial behavior and positively associated with prosocial behavior. Regulation is fundamentally related to lability/negativity because children who are more emotionally labile have stronger emotions that are more difficult to regulate. Nevertheless, we can distinguish between processes involved in the elicitation of emotions (lability/negativity) and processes involved in the management of emotions (regulation).

These two components of emotion regulation may be intimately linked with socially avoidant children’s adjustment. According to the *goodness-of-fit* theory (Thomas and Chess, 1977), child socialization effects depend on child own temperamental characteristics (Rothbart and Ahadi, 1994). Some characteristics, such as social avoidance, may represent a risk factor in development. Certain social emotional factors may serve as a protective factor that reduces the risk and protects children with such characteristics from developing adjustment difficulties (Penela et al., 2015). In contrast, lack of those factors may exacerbate children’s risks for developing maladaptive functioning. For instance, socially avoidant children tend to respond to stressful social situations with greater reactivity and emotional volatility (Kagan et al., 1987), but possessing greater emotion regulation skills helps reduce emotional reactions to stressful, anxiety-provoking situations (Campbell-Sills and Barlow, 2007; Werner and Gross, 2010). Similarly, deficits in emotion regulation ability and heightened lability may serve to reinforce socially avoidant children’s inappropriate social skills and evoke peer rejection and exclusion (Rubin et al., 1995). Specifically, being less capable of regulating negative emotions during social interaction may contribute to sustained anxiety. Indeed, previous studies have shown that the ability to regulate negative emotions is important for socially withdrawn children (e.g., Rubin et al., 1995; Coplan et al., 2001; Hipson et al., 2019). For example, Penela et al. (2015) found that emotion regulation decreased the risk for peer problems and social competence deficits among children with high levels of behavioral inhibition. Similarly, Henderson (2010) reported that self-reported shyness was associated with social anxiety and a negative attribution style, but only among children with poorer emotion regulation skills.

Moreover, Chinese culture attaches importance to interdependence and social harmony (Oyserman et al., 2002). Thus, Chinese parents and teachers endorse values and socialize their children to foster cooperation, suppress anger and aggression (Chao, 2000; Wang et al., 2006). And children’s emotion regulation skills are highly valued and reinforced. Thus, emotion regulation may be helpful in mitigating maladjustment among Chinese socially avoidant children. These links notwithstanding, it is not yet known whether lability/negativity and regulation play similar moderating roles among Chinese children.

### The Present Study

**FIGURE 1:**
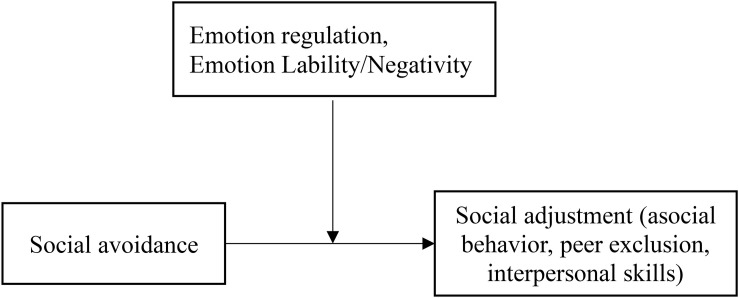
The hypotheses model.

The purpose of this study was to explore the moderating role of emotion regulation and emotion lability in the associations between social avoidance and adjustment difficulties among Chinese young children. Based on previous studies that Chinese socially avoidant was associated with negative adjustment (Coplan et al., 2016; Sang et al., 2018). We expected that social avoidance would be positively associated with asocial behavior, peer exclusion, internalizing problems, and negatively associated with interpersonal skills among Chinese children. Moreover, as previous studies have shown that deficits in emotion regulation ability and heightened lability may serve to reinforce socially avoidant children’s inappropriate social skills and evoke peer rejection and exclusion (Rubin et al., 1995). We hypothesized that components of emotion regulation (i.e., emotion regulation and emotion lability/negativity) would moderate socially avoidant children’s adjustment difficulties. Specifically, we expected that emotion lability would exacerbate socially avoidant children’s risk for social skills deficits and peer exclusion whereas regulation would mitigate these associations (see Figure 1).

## Materials and Methods

### Participants

Participants were *N* = 194 children (102 boys, 92 girls, *M*_*age*_ = 70.82 months, SD = 5.40) recruited from two public kindergartens in Shanghai, People’s Republic of China. In China, children attend kindergarten for 3 years and are typically grouped by age (e.g., junior class: ages 3–4 years; middle class: aged 4–5 years; senior class: ages 5–6 years). All children were of Han ethnicity, which represents over 97% of China’s population.

Nearly 13% of the mothers and 13% of the fathers had completed high school; 20% of the mothers and 20% of the fathers had completed junior college; 52% of the mothers and 40% of the fathers had earned a bachelor’s degree; and 15% of the mothers and 27% of the fathers had earned a postgraduate degree. Maternal and paternal scores were averaged to create a broader measure of parental education (with higher scores representing higher education).

### Procedure

The present study was reviewed and approved by the ethics review board of Shanghai Normal University. All students in each of the participating classes were invited to participate in the study. Written consent was obtained from parents of all children through the school. The participation rate was 98%. Mothers rated their children’s social avoidance and teachers completed measures of children’s social adjustment and emotion regulation.

### Measures

#### Maternal Ratings

Mothers completed the Chinese version of Child Social Preference Scale (CSPS; Coplan et al., 2004; Li et al., 2016). Of particular interest was the subscale assessing *social avoidance*, which comprises four items (e.g., “My child actively avoids playing with other children”; α = 0.76). Given the shared conceptual overlap and similar patterns of adjustment among withdrawn Chinese youth, it is important to control for any shared variance with shyness and unsociability when exploring the implications of social avoidance among Chinese children (e.g., Sang et al., 2018). As such, mother also completed the *shyness* subscale, which comprises seven items (e.g., “Although he/she appears to desire to play with others, my child is sometimes anxious about interacting with other children”; α = 0.89), and *unsociability* subscale, which comprises four items (e.g., “My child is just as happy to play quietly by his/herself than to play with a group of children”; α = 0.67) rated on a five-point scale (from 1 = “not at all” to 5 = “a lot”). These items were aggregated to create the social avoidance score, with higher scores indicating higher levels of social avoidance. The CSPS has been shown to be reliable and valid in young Chinese children (Li et al., 2016).

#### Teacher Ratings

Teachers completed the Chinese adaptation of the Emotion Regulation Checklist (ERC; Shields and Cicchetti, 1997). The ERC scale comprises 21 items, consisting of two dimensions: *Emotion Regulation* (8 items, e.g., “He/She is a cheerful child”; α = 0.86) and *Lability/Negativity* (13 items, e.g., “He/She is prone to angry outbursts/tantrums easily”; α = 0.91). Items were rated on a four-point scale (from 1 = “never” to 4 = “almost always”). The ERC has been shown to be reliable and valid in young Chinese children (Zhu et al., 2020).

Teachers also completed the Chinese version of Child Behavior Scale (CBS; Ladd and Profilet, 1996; Zhu et al., 2018). Of particular interest were subscales assessing *asocial behavior* (six items, e.g., “likes to be alone, withdraws from peer activities”; ɑ = 0.87), and *peer exclusion* (seven items, e.g., “not welcomed by other children”; ɑ = 0.86). Items were rated on a three-point scale (from 1 = “doesn’t apply” to 3 = “certainly applies”). The CBS has been shown to be reliable and valid in young Chinese children (Zhu et al., 2018).

Finally, teachers completed the Chinese version of Social Skills Teacher Rating System (SSTRS; Gresham and Elliott, 1990; Zhu et al., 2017a). We were particularly interested in the subscales assessing *interpersonal skills* (11 items, e.g., “Easy to make friends”; ɑ = 0.93). Items were rated on a three-point scale (from 0 = “never” to 2 = “always”). The SSTRS has been shown to be reliable and valid in young Chinese children (Zhu et al., 2017b).

### Analytical Strategy

We used SPSS 22 software for data analysis. Preliminary analyses included a series of *t*-tests to explore gender differences and correlations among study variables. To explore moderating effect, we used the PROCESS macro (Hayes, 2012) (Model 1) with non-parametric bootstrapping with 1,000 resamples. We used a 95% bias-corrected confidence interval (CI) to test for significant moderations (Preacher and Hayes, 2008).

## Results

### Preliminary Analyses

Descriptive statistics and correlations for all study variables are displayed in Table 1. Results from *t*-tests indicated no gender differences in lability/negativity (*M*_*boy*_ = 1.86, *SD* = 0.54; *M*_*girl*_ = 1.74, *SD* = 0.56, *t* = 1.42, *p* = 0.76), emotion regulation (*M*_*boy*_ = 2.66, *SD* = 0.60; *M*_*girl*_ = 2.78, *SD* = 0.58, *t* = −1.41, *p* = 0.16), and mother-rated child shyness (*M*_*boy*_ = 12.58, *SD* = 4.13; *M*_*girl*_ = 12.71, *SD* = 4.52, *t* = −0.21, *p* = 0.83), unsociability (*M*_*boy*_ = 7.54, *SD* = 2.60; *M*_*girl*_ = 7.11, *SD* = 2.51, *t* = 1.82, *p* = 0.24), and social avoidance (*M*_*boy*_ = 5.58, *SD* = 1.71; *M*_*girl*_ = 5.65, *SD* = 1.96, *t* = −0.26, *p* = 0.80). Similarly, there were no gender differences in teacher-rated asocial behavior (*M*_*boy*_ = 1.28, *SD* = 0.41; *M*_*girl*_ = 1.20, *SD* = 0.36, *t* = 1.39, *p* = 0.12), peer exclusion (*M*_*boy*_ = 1.25, *SD* = 0.45; *M*_*girl*_ = 1.16, *SD* = 0.30, *t* = 1.71, *p* = 0.09) and interpersonal skills (*M*_*boy*_ = 1.55, *SD* = 0.47; *M*_*girl*_ = 1.64, *SD* = 0.40, *t* = −1.44, *p* = 0.15). Parental education was significantly and positively correlated with social avoidance. Accordingly, we controlled for parental education in subsequent analyses.

**TABLE 1:** Descriptive statistics and inter-correlations for all study variables (*N* = 194).

	1	2	3	4	5	6	7	8	9	10
1. Shyness	−									
2. Unsociability	0.61***	−								
3. Social avoidance	0.68***	0.58***	−							
4. Gender	0.05	–0.06	0.01	−						
5. Parental education	0.10	0.13	0.18*	0.05	−					
6. Asocial behavior	0.20**	0.13	0.17*	–0.09	0.05	−				
7. Peer exclusion	0.12	0.12	0.14	–0.12	0.03	0.74***	−			
8. Interpersonal skills	−0.19**	–0.14	–0.13	0.09	0.09	−0.48***	−0.46***	−		
9. Lability\Negativity	–0.10	–0.08	–0.09	–0.10	–0.05	0.20**	0.28***	−0.26***	−	
10. Emotion regulation	0.01	–0.06	0.02	0.10	0.20**	−0.24**	−0.29***	0.27***	−0.39***	−
*M*	1.81	1.82	1.41	1.47	5.50	1.24	1.21	1.59	1.80	2.72
*SD*	0.61	0.64	0.46	0.50	1.68	0.38	0.39	0.44	0.55	0.59

*p < 0.05, **p < 0.01, ***p < 0.001.

As indicated in Table 1, social avoidance was significantly and positively associated with asocial behavior. Emotion regulation was also significantly and negatively associated with asocial behavior and peer exclusion. In addition, emotion regulation was positively associated with interpersonal skills. In contrast, emotional lability/negativity was also significantly and positively associated with asocial behavior, peer exclusion, and negatively associated with interpersonal skills.

### Social Avoidance, Emotion Regulation, and Social Adjustment

Classroom intraclass correlations (ICC) were less than 0.07 and non-significant for all the variables, indicating no cluster effects for the classroom in the present study. We tested the moderating effects of emotion regulation and lability in relation between social avoidance and outcome variables (i.e., asocial behavior, peer exclusion, interpersonal skills), while controlling for parental education. Results are displayed in Table 2. These findings were largely consistent with the correlational analyses (despite the additional control variables). Of particular interest, there were significant interaction effects between social avoidance and emotion regulation were also found in relation to asocial behavior, peer exclusion and interpersonal skills. Also the significant interaction effects between social avoidance and lability/negativity in relation to asocial behavior and peer exclusion.

**TABLE 2:** Effects of social avoidance, emotion regulation and lability/negativity (controlling for parental education) in relation to indices of social adjustment.

Social adjustment variables				
Predictor	*B*	*SE*	*t-value*	*95% CI*
**Asocial behavior**				
Shyness	0.16	0.10	1.68	[−0.03, 0.36]
Unsociability	–0.05	0.09	–0.52	[−0.22, 0.13]
Social avoidance	0.12	0.10	0.23	[−0.08, 0.31]
Emotion regulation	–0.23	0.07	−3.30**	[−0.37, −0.09]
Avoidance × ER	–0.20	0.07	−2.88**	[−0.32, −0.06]
**Peer exclusion**				
Shyness	0.04	0.10	0.36	[−0.15, 0.23]
Unsociability	0.01	0.09	0.08	[−0.17, 0.18]
Social avoidance	0.14	0.10	1.40	[−0.06, 0.33]
Emotion regulation	–0.28	0.07	−4.07***	[−0.42, −0.15]
Avoidance × ER	–0.18	0.07	−2.74**	[−0.31, −0.05]
**Interpersonal skills**				
Shyness	–0.18	0.10	–1.79	[−0.38, 0.02]
Unsociability	–0.01	0.09	–0.08	[−0.19, 0.17]
Social avoidance	–0.05	0.10	–0.48	[−0.24, 0.15]
Emotion regulation	0.24	0.07	3.34**	[0.10, 0.37]
Avoidance × ER	0.15	0.07	2.20*	[0.02, 0.28]
**Asocial behavior**				
Shyness	0.18	0.10	1.81	[−0.02, 0.38]
Unsociability	–0.01	0.09	–0.09	[−0.19, 0.17]
Social avoidance	0.08	0.10	0.80	[−0.12, 0.27]
Lability\Negativity	0.26	0.07	3.65***	[0.12, 0.39]
Avoidance × LN	0.18	0.08	2.45*	[0.04, 0.33]
**Peer exclusion**				
Shyness	0.05	0.10	0.52	[−0.14, 0.25]
Unsociability	0.06	0.09	0.61	[−0.12, 0.23]
Social avoidance	0.10	0.10	1.03	[−0.09, 0.29]
Lability\Negativity	0.33	0.07	4.67***	[0.19, 0.46]
Avoidance × LN	0.16	0.07	2.19*	[0.02, 0.31]
**Interpersonal skills**				
Shyness	–0.19	0.10	–1.85	[−0.38, 0.01]
Unsociability	–0.05	0.09	–0.59	[−0.23, 0.13]
Social avoidance	–0.02	0.10	–0.16	[−0.21, 0.18]
Lability\Negativity	0.28	0.07	−4.05***	[−0.42, −0.15]
Avoidance × LN	–0.05	0.08	–0.61	[−0.19, 0.10]

*p < 0.05, **p < 0.01, ***p < 0.001.

**FIGURE 2:**
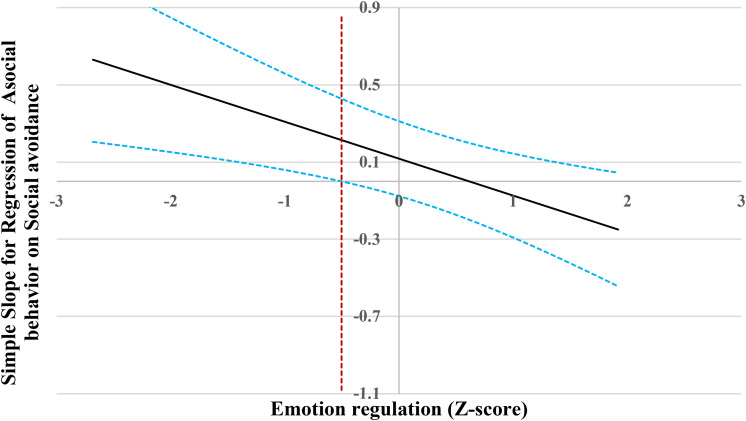
Johnson-Neyman regions of significance and confidence bands for mother-rated social avoidance along emotion regulation in relation to asocial behavior. Solid diagonal line represents the regression coefficient for social avoidance along emotion regulation. Dashed diagonal blue lines are confidence bands—upper and lower bounds of 95% confidence interval for social avoidance regression coefficient along emotion regulation. The dashed vertical red line indicates the point along emotion regulation at which the social avoidance regression coefficient transitions from statistical significance (left of dashed vertical line) to non-significance (right of dashed vertical line). The value of the dashed vertical line is –0.50.

**FIGURE 3:**
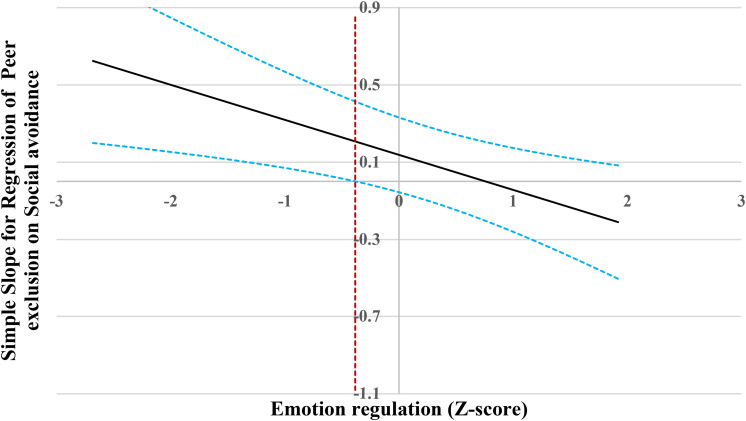
Johnson-Neyman regions of significance and confidence bands for mother-rated social avoidance along emotion regulation in relation to peer exclusion. Solid diagonal line represents the regression coefficient for social avoidance along emotion regulation. Dashed diagonal blue lines are confidence bands—upper and lower bounds of 95% confidence interval for social avoidance regression coefficient along emotion regulation. The dashed vertical red line indicates the point along emotion regulation at which the social avoidance regression coefficient transitions from statistical significance (left of dashed vertical line) to non-significance (right of dashed vertical line). The value of the dashed vertical line is –0.38.

**FIGURE 4:**
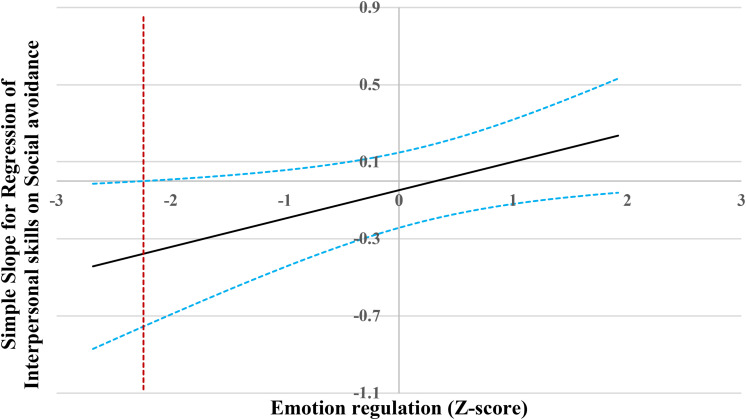
Johnson-Neyman regions of significance and confidence bands for mother-rated social avoidance along emotion regulation in relation to interpersonal skills. Solid diagonal line represents the regression coefficient for social avoidance along emotion regulation. Dashed diagonal blue lines are confidence bands—upper and lower bounds of 95% confidence interval for social avoidance regression coefficient along emotion regulation. The dashed vertical red line indicates the point along emotion regulation at which the social avoidance regression coefficient transitions from statistical significance (left of dashed vertical line) to non-significance (right of dashed vertical line). The value of the dashed vertical line is –2.24.

**FIGURE 5:**
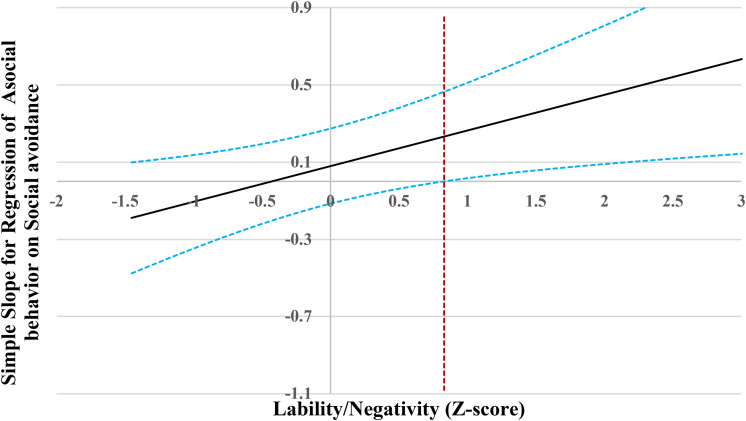
Johnson-Neyman regions of significance and confidence bands for mother-rated social avoidance along lability/negativity in relation to asocial behavior. Solid diagonal line represents the regression coefficient for social avoidance along lability/negativity. Dashed diagonal blue lines are confidence bands—upper and lower bounds of 95% confidence interval for social avoidance regression coefficient along lability/negativity. The dashed vertical red line indicates the point along lability/negativity at which the social avoidance regression coefficient transitions from non-significance (left of dashed vertical line) to statistical significance (right of dashed vertical line). The value of the dashed vertical line is 0.83.

**FIGURE 6:**
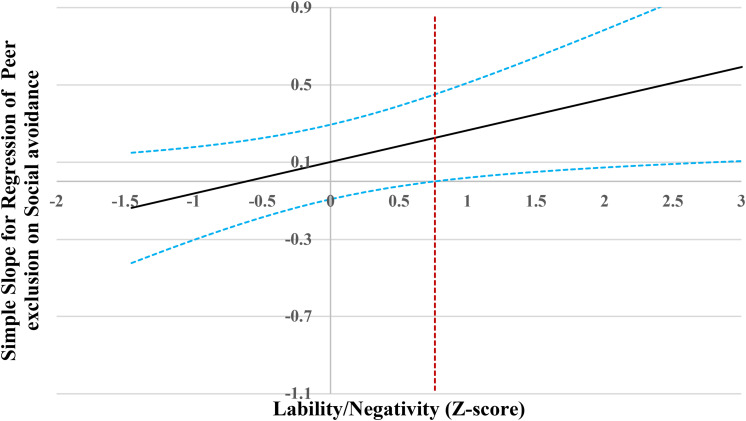
Johnson-Neyman regions of significance and confidence bands for mother-rated social avoidance along lability/negativity in relation to peer exclusion. Solid diagonal line represents the regression coefficient for social avoidance along lability/negativity. Dashed diagonal blue lines are confidence bands—upper and lower bounds of 95% confidence interval for social avoidance regression coefficient along lability/negativity. The dashed vertical red line indicates the point along lability/negativity at which the social avoidance regression coefficient transitions from non-significance (left of dashed vertical line) to statistical significance (right of dashed vertical line). The value of the dashed vertical line is 0.76.

Following suggestions by Hayes and Matthes (2009), we used the Johnson-Neyman (J-N) technique (Johnson and Fay, 1950) to further probe the significant interactions (all the predictors were standardized for the analyses). This technique allowed us to estimate a region of significance for the simple slope of a predictor conditioned on the value of the continuous moderator. The results are presented visually in Figures 2–6.

For the prediction of asocial behavior (Figure 2), when emotion regulation level was lower than −0.50 SD, social avoidance was significantly and positively associated with asocial behavior. However, when emotion regulation level was higher than −0.50 SD, social avoidance was no longer associated with asocial behavior.

For the prediction of peer exclusion (Figure 3), when emotion regulation level was lower than −0.38 SD, social avoidance was significantly and positively associated with peer exclusion. However, when emotion regulation level was higher than −0.38 SD, social avoidance was no longer associated with peer exclusion.

For the prediction of interpersonal skills (Figure 4), when emotion regulation level was lower than −2.24 SD, social avoidance was significantly and negatively associated with interpersonal skills. However, when emotion regulation level was higher than −2.24 SD, social avoidance was no longer associated with interpersonal skills.

When lability/negativity level was lower than 0.83 SD, social avoidance was not associated with asocial behavior (Figure 5). However, when lability\negativity level was higher than 0.83 SD, social avoidance was positively associated with asocial behavior.

When lability/negativity level was lower than 0.76 SD, social avoidance was not associated with peer exclusion (Figure 6). However, when lability/negativity level was higher than 0.76 SD, social avoidance was significantly and positively associated with peer exclusion.

## Discussion

Although previous studies provided meaningful information of the associations between social avoidance and social, school, and psychological adjustment difficulties across different contexts (Ding et al., 2015; Coplan et al., 2016), the possible moderating role of social emotional factors involved in this relationship remain unexplored, specifically in Chinese culture. Thus, the primary goal of present study was to explore the moderating role of emotion regulation and emotion lability/negativity in the relations between social avoidance and socio-emotional adjustment outcomes in the context of contemporary urban China. We specifically examined under which conditions social avoidance is associated with socio-emotional adjustment difficulties. In general, our results revealed that emotion regulation moderate the association between social avoidance and asocial behavior, interpersonal skills.

Overall, emotion regulation was negatively associated with asocial behavior and peer exclusion and was positively associated with interpersonal skills. In contrast, emotional lability/negativity was positively associated with asocial behavior, peer exclusion, and negatively associated with interpersonal skills. These results are consistent with previous studies showing that emotion regulation is associated with higher levels of social competence and peer acceptance (e.g., Kochanska et al., 1997; Eisenberg et al., 2004) and that emotion lability/negativity is associated with less social competence and more externalizing problems (Eisenberg et al., 1995, 2004; Kim and Deater-Deckard, 2011). However, social avoidance was not related to the child’s socio-emotional adjustment difficulties (e.g., peer exclusion and interpersonal skills). Researchers argued that children are more self-focused during this age. Therefore, solitary behavior is quite common in the preschool (Coplan et al., 2004). Thus, social avoidant child might not display social difficulties during kindergarten. Moreover, it is also possible due to observational bias (e.g., all socio-emotional adjustment outcomes were rated by their teachers). Thus, it would be important to replicate the present findings among Chinese young children and using multiple resources to collect data. Consistent to our expectations, emotion lability/negativity moderated the association between social avoidance and asocial behavior, peer exclusion. Specifically, social avoidance was only positively associated with asocial behavior and peer exclusion among children higher in emotion lability/negativity. Thus, emotion lability/negativity may reinforce socially avoidant children’s inappropriate social skills and evoke peer rejection and exclusion in Chinese culture. According to social anxiety cognition model (Leary et al., 1988), when socially anxious individuals tend to assume that their interaction partner will perceive them negatively (Rapee and Heimberg, 1997; Cole and McCroskey, 2003; Goldin et al., 2009). Children high in emotional lability/negativity also experience large mood swings and are easily frustrated. This has implications for how they interact with and are perceived by others, perhaps leading to greater peer rejection and exclusion (Salzer Burks et al., 1999).

Consistent with our hypotheses, emotion regulation also moderated the association between social avoidance and asocial behavior and interpersonal skills. Specifically, social avoidance was only positively associated with asocial behavior when children who reported lower in emotion regulation. Similarly, social avoidance was only negatively associated with interpersonal skills among children who reported lower in emotion regulation skills. These findings suggest that in emotion regulation may act as a buffering effect in the link between social avoidance and social adjustment issues among Chinese children as has been suggested in Western samples (e.g., Rubin et al., 1995; Coplan et al., 2001; Penela et al., 2015).

It is not surprising that emotion regulation of the socially avoidant children in our study was associated with less asocial behavior and better interpersonal skills. Social situations are particularly stressful to children who are social avoidant, they may worry about receiving negative feedback from peers, and are thus prone to more negative emotions, such as fear and sadness (Han and Huang, 2010). Emotion regulation, which involves selecting and employing strategies for dealing with stressful social situations, may help these children reduce negative emotion expression and establish better relationships with peers (Cohen and Mendez, 2009; Monopoli and Kingston, 2012). Indeed, emotion regulation may aid in children’s communicative skills. A core aspect of social skills is demonstrating appropriate behavioral engagement (e.g., turn-taking) (Dunn and Brown, 1994). In contrast, dysregulation could lead to socially inappropriate behavior and impair children’s abilities to successfully interact with peers, which may, in turn, lead to more social withdrawal.

### Limitations and Future Direction

The present study first explored the associations between social avoidance, emotion regulation, and socio-emotional adjustment outcomes in the cultural context of contemporary urban China. Despite the contribution of this research to the extant literature, there are some limitations that should be noted. First, the present study was correlational in nature, which negates our ability to establish causal links and the direction of effects between the constructs under investigation. It is possible, for example that negative peer experiences may promote social avoidance over time (Bowker and Raja, 2011). To address these limitations, we recommend that future research adopt a longitudinal design which will allow for testing these effects (MacKinnon, 2008).

Moreover, although our data were collected from multiple resources (i.e., parent- and teacher-reports), there still were some potential methodological concerns with our assessment. For example, children’s emotion regulation ability and all socio-emotional adjustment outcomes were rated by their teachers. Furthermore, children were all nested within classrooms, which means that children from one class were rated by the same teacher. Thus, there is a clear informant confound. It would be important to replicate the present findings using multiple levels of assessment (e.g., observations in laboratory paradigms and in naturalistic contexts, self-report). Also, in further study, we would conduct child interviews for the indices of their social adjustment.

Furthermore, we tested a conceptual model in which emotion regulation were proposed as moderators in the links between children’s social avoidance and adjustment outcomes. However, other theoretical interpretations may be plausible. For example, researchers argued that socially avoidant children are more likely to have difficulty regulating emotions because they experience heightened emotional reactivity (Hane et al., 2008). In addition, deficits in emotion regulation may explain the link between social avoidance and social adjustment difficulties. For example, Hipson et al. (2019) found that emotion regulation mediated associations between shyness and subsequent prosocial and socially withdrawn behaviors among preschoolers. Similarly, Penela et al. (2015) found that child inhibition control predicted less active emotion regulation, which subsequently predicted social competence. Thus, future study should explore the mediating role of emotion regulation in the relation between child social avoidance and adjustment difficulties. Also, the current study only examined Chinese children’ development. Future study could conduct Western-Asian culture compassion study.

Despite the limitations and weaknesses, the results of the present study constitute a significant contribution to our understanding of relations between social avoidance and adjustment outcomes and the role of emotion regulation in today’s urban Chinese children. And our findings may have practical implications for early intervention program for socially avoidant children. For example, teachers and parents need to remember that emotion regulation plays a very important role in the development of social competence. Thus, practitioners and educators should help socially avoidant children develop their emotion regulation skills, especially in Chinese culture. Specifically, parents and teachers could consider organizing reading activities related to emotion regulation (e.g., read picture books on the topic of emotion regulation). Parents could also encourage their children to participate in sports activities to improve their interpersonal skills, experience positive emotion, and enhance their self-esteem.

## Data Availability Statement

The data and materials used during the current study are available from the corresponding author (YL) on reasonable request.

## Ethics Statement

The studies involving human participants were reviewed and approved by Shanghai Normal University. Written informed consent to participate in this study was provided by the participants’ legal guardian/next of kin.

## Author Contributions

JZ and YL conceived of the presented idea. JZ analyzed the data and wrote up the methods and results section. BX and WH wrote up the first draft. JZ verified the analytical methods. YL, RC, and CY supervised the findings of this work. All authors discussed the results and contributed to the final manuscript.

## Conflict of Interest

The authors declare that the research was conducted in the absence of any commercial or financial relationships that could be construed as a potential conflict of interest.
